# Translation and validation of a simplified Chinese version of the psychosocial assessment tool

**DOI:** 10.1186/s12885-024-11947-x

**Published:** 2024-02-16

**Authors:** Jun Kou, Ruiqi Wang, Yuxin Tang, Yi Tang, Yang Gao

**Affiliations:** Department of Ultrasound, National Clinical Research Center for Child Health and Disorders, Ministry of Education Key Laboratory of Child Development and Disorders.(Chongqing Key Laboratory of Pediatrics), Children’s Hospital of Chongqing Medical University, Chongqing, 400010 China

**Keywords:** Oncology, Families, Pediatrics, Adaptation, Translation

## Abstract

**Background:**

The Psychosocial Assessment Tool (PAT2.0) is widely used to assess psychosocial risk in families of children with cancer. Our study aims to apply PAT2.0 to Chinese patients and assess the reliability, content validity, and construct validity of the Chinese version.

**Methods:**

A total of 161 participants completed the study, each with only one child diagnosed with cancer. Psychometric evaluations, including internal consistency, score distribution, test-retest reliability, and construct validity, were conducted.

**Results:**

Cronbach’s alpha values ranged from 0.732 to 0.843, indicating good internal consistency. Additionally, intraclass correlation coefficient values ranged from 0.869 to 0.984, indicating excellent test-retest reliability. The Simplified Chinese version of PAT2.0 demonstrated high construct validity in factor analyses and correlations with the General Functioning Subscale of the Family Assessment Device.

**Conclusion:**

The translation process of the Chinese version of PAT2.0 was successful, proving its applicability for psychosocial evaluation and interventions in families of children with cancer in China.

## Background

Childhood cancer primarily results from genetic factors, although specific unhealthy lifestyle habits and environmental influences have also been associated with the disease, posing a significant health threat to children [1]. Children diagnosed with cancer may undergo pain and trauma, impacting their families and potentially leading to severe mental health conditions [2, 3]. Timely intervention can mitigate the progression of mental health issues and potentially enhance the prognosis for children with cancer. Unfortunately, most families dealing with childhood cancer lack essential psychological support [2, 3]. While the incidence of childhood cancer is lower than in adulthood [4], the latest survey indicates a notable increase in the occurrence of childhood cancer in China [5]. Given China’s large population, a substantial number of children endure the pain and distress caused by cancer. The psychosocial challenges stemming from childhood cancer diagnoses and their effects on families have become a major concern for Chinese society.

The Psychosocial Assessment Tool (PAT2.0) proves valuable as a screening tool for assessing the psychosocial risks faced by families with children suffering from cancer. This tool enables medical and allied health providers to objectively evaluate risks arising from changes in family functions [6]. Many screening methods concentrate on severe mental illness and may not effectively identify individuals with mild mental illness at risk of psychosocial distress [7]. PAT 2.0, a family-centered and family-focused program grounded in scientific research and clinical experience, is specifically designed to address multiple risk factors contributing to psychosocial distress [8]. PAT2.0 has been successfully translated into Dutch [9], Japanese [10], and Turkish [11], with demonstrated reliability and validity. Presently, there is no research available on the translation of PAT2.0 into Chinese, and no validated tool exists to systematically assess and screen for psychosocial problems in families of children with cancer in China. To address this gap, we cross-culturally adapted PAT2.0 into Simplified Chinese (SC-PAT2.0) and evaluated its reliability and validity in assessing psychosocial risks faced by families of children with cancer in Mainland China.

## Methods

This study aimed to develop an instrument to evaluate the psychosocial status of patients to furnish valuable insights for clinical practice and research. Initially, the researchers translated the Psychosocial Assessment Tool into a Simplified Chinese version, ensuring cultural adaptation for applicability and accuracy in the Chinese cultural context. Subsequently, the researchers validated the Simplified Chinese version of the psychosocial assessment tool to guarantee its accuracy and reliability.

### Translation and cross‑cultural adaptation

Following previously published guidelines [12], the PAT 2.0 underwent translation into Simplified Chinese in a five-step process: initial translation, synthesis of translations, back translation, expert committee review, and pretesting. A detailed description of this process is available in other publications [13, 14]. Content evaluation involved a native Chinese physician with an English-speaking background and two pediatric oncologists, assessing the accuracy, clarity, logic, and appropriateness of the questionnaire for the target audience. The final version of SC-PAT2.0 resulted from the collective opinions of all research members.

### Patients and data acquisition

Family members of children with cancer were recruited from both outpatient and inpatient settings, adhering to specific criteria: (1) families with a single child aged 2 to 18 diagnosed with cancer; (2) participants aged over 18 when completing the questionnaire; (3) individuals capable of reading and speaking Chinese. Exclusion criteria included: (1) severe mental disorders, including psychiatric conditions; (2) children with cancer whose family lacked a history of chronic or life-threatening diseases; (3) family members who had already participated in the study.

Before participation, all involved individuals carefully read and signed an informed consent form approved by the Ethics Committee. On the first day of inclusion, participants provided demographic information and independently completed two scales (SC-PAT2.0, the Chinese version of the General Functioning Subscale of the Family Assessment Device [FAD-GF]) in a quiet meeting room. Four to seven days later, participants retook the SC-PAT2.0 to assess the test-retest reliability of the scale. Children from the participant’s family undergoing treatment that might impact them during the second completion of the questionnaire were excluded from the study.

### Scales

PAT2.0 is a concise screening tool utilized in families with children affected by cancer to evaluate the psychological risks within the family context [6]. Comprising seven subscales, PAT2.0 assesses family structure and resources, family social support, family problems, parent stress reactions, family beliefs, child problems, and sibling problems [6]. PAT2.0 was developed for families of children with cancer. A higher PAT2.0 score indicates a heightened psychological risk within the family [6].

FAD-GF is a condensed version of the McMaster Family Assessment Device, introduced by Epstein in 1983 [15]. It features twelve questions, each scored from 1 to 4, with higher scores indicating poorer family functioning. Overall family functioning is assessed by calculating the total score [16].

Both questionnaires assess family function through self-report measures. The FAD-GF is a versatile tool for evaluating households in various contexts, and the Chinese version of the FAD-GF has been demonstrated good reliability and validity [17]. In contrast, PAT2.0 is a psychosocial assessment tool specifically designed to evaluate children with cancer and their families, providing more specific and targeted results that help to better understand the needs and challenges of these families.

### Psychometric assessments and statistical analysis

The evaluation of SC-PAT2.0 focused on reliability, content validity, and construct validity.

Reliability testing involves assessing internal consistency and test-retest reliability [18]. Internal consistency, indicating the extent of interaction between items, is primarily evaluated through the Cronbach’s α value of the scale. An α value exceeding 0.9 signifies excellent internal consistency, while values exceeding 0.8 and 0.7 are considered indicative of good and acceptable internal consistency, respectively [19]. To assess test-retest reliability, family members completed SC-PAT 2.0 twice within 4–7 days, ensuring the health status of cancer-afflicted children in their family remained unchanged between the first and second tests [20]. The intraclass correlation coefficient (ICC), derived from two-way ANOVA in random effects models, is a commonly used measure for test-retest reliability [21]. An ICC greater than 0.9 and 0.8 signifies excellent and good reliability, respectively [22]. Additionally, to investigate measurement errors, the standard error of measurement (SEM) and the minimal detectable change (MDC) were calculated. Measurement errors encompass randomness and systematic errors unrelated to actual changes in the tested structure, arising from patient ratings [23]. The SEM is computed as SD×√(1-ICC). The MDC, representing the minimum individual change in scores and considered the true change, was calculated as SEM×1.96×√2/√n at the group level and SEM×1.96×√2 at the individual level. Systematic errors of the scale can be further observed by depicting the Bland-Altman diagram [22].

Content validity is primarily evident in assessing item relevance and comprehensiveness [22]. Currently, the three most commonly used comprehensive project evaluation indices are patient feedback, response rate, and ceiling/floor effect [22]. If the ceiling/floor effect of a scale is less than 15%, patient feedback exceeds 95%, and there is no difficulty reported in completing the scale, then the scale is considered highly comprehensive.

Construct validity was evaluated through factor analysis and correlation calculations between SC-PAT2.0 scores and FAD-GF scores. Initially, an exploratory factor analysis on SC-PAT2.0 was conducted, using the Kaiser-Meyer-Olkin (KMO) measurement and Bartlett test for sphericity to assess sampling adequacy [24]. The dataset is considered suitable for factor analysis only when Bartlett’s sphericity test is significant (*P* < 0.05) and KMO > 0.60 [25]. Given that PAT2.0 has multiple dimensions, confirmatory factor analysis (CFA) was performed on SC-PAT2.0 to evaluate model fit and parameter estimation. The correlation between SC-PAT2.0 scores and FAD-GF scores was determined using Pearson’s correlation coefficient, with results categorized as ‘excellent’ (r > 0.8), ‘very good’ (r = 0.61–0.80), ‘moderate’ (r = 0.41–0.60), ‘fair’ (r = 0.21–0.40), and ‘poor’ (*r* < 0.20 or *p* > 0.05) [26].

### Ethical statement

All procedures performed in this study involving human participants were carried out in accordance with the 1964 Helsinki Declaration and its later amendments or comparable ethical standards. All participants read and signed informed consent, and this clinical study obtained the approval of the ethics committee of our hospital(CHCMU-XJS-2019-20).

## Results

### Patients

From April 2023 to October 2023, 161 parents of patients meeting screening criteria fully participated in the study. Table 1 provides detailed demographics of these participants. The study included 79 mothers, 65 fathers, 9 grandfathers, and 8 grandmothers as participants.

**Table 1:** Demographic and clinical characteristics of participants

	Child characteristics		Parent characteristics	
	Number	%	Number	%
Gender Female Male Age (years) Range Mean ± SD Diagnosis Leukemia and lymphoma Soft-Tissue and Bone Tumors CNS tumor Other Solid Tumors	68 93 2–18 7.49 ± 4.29 28 45 39 49	42.24 57.76 17.39 27.95 24.22 30.43	87 74 20–50 31.66 ± 6.19	54.04 45.96

SD standard deviation, CNS Central Nervous System

### Translation and cross‑culture adaptation process

The forward and backward translation of SC-PAT2.0 proceeded smoothly. No modifications were made to SC-PAT2.0 due to points of incomprehension, and no patient’s family member reported difficulty in understanding the project.

### Validity

**Fig. 1:**
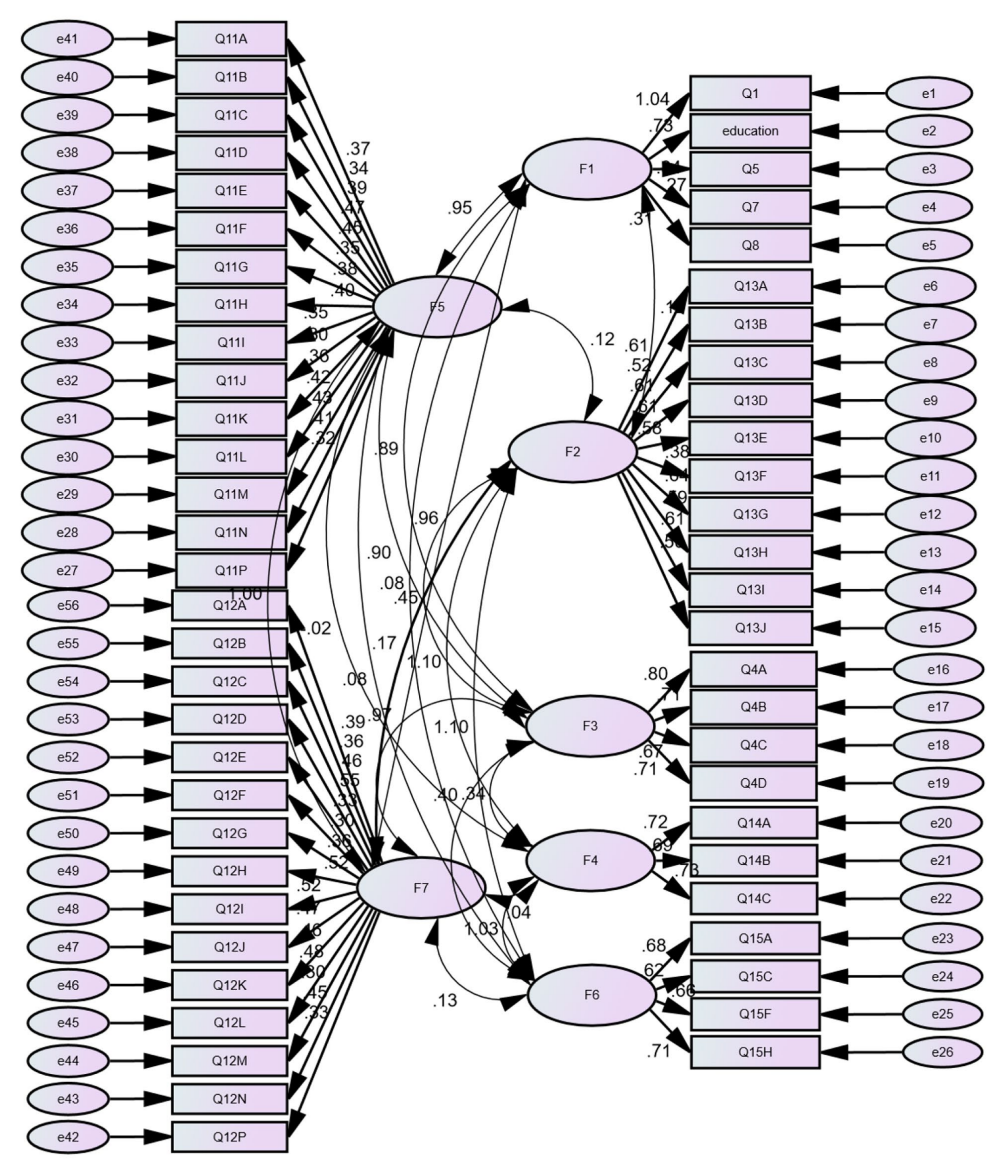
Standardized seven-factor structural model of SC-PAT 2.0. F1 (Structure/resources, 5 items), F2 (Family problems, 10 items), F3 (Social support, four items), F4 (Stress reaction, three items), F5 (Child problems, 15 items), F6 (Family beliefs, 4 items), F7 (Sibling problems, 15 items)

Participants reported no difficulty in understanding the content of SC-PAT2.0. The distribution of SC-PAT2.0 scores indicates no floor effect (0%~6.83% [< 15%]) or ceiling effect (0%~1.86% [< 15%]) (Table 2). These findings suggest that SC-PAT2.0 possesses good content validity. The Bartlett sphericity test was significant (*p* < 0.001), and the KMO index was 0.842, surpassing the acceptable minimum value of 0.6. Consequently, the matrix is suitable for factor extraction. Model fit indices were satisfactory: 2.009 for Chi-Square Minimum/Degrees of Freedom, 0.079 for Root Mean Square Error of Approximation, and 0.650 for Incremental Fit Index. The results of confirmatory factor analysis (CFA) are depicted in Fig. 1. Table 3 presents the pertinent data for the evaluation of construct validity for SC-PAT2.0.

**Table 2:** The reliability of the SC-PAT 2.0

	Cronbach’s α(95% CI)	First test^a^	Second test^a^	ICC (95% CI)	SEM	MDC^b^	MDC^c^
Total	0.818(0.771–0.858)	2.860 ± 1.277	2.874 ± 1.075	0.984(0.979–0.989)	0.161	0.035	0.448
Structure/resources	0.771(0.709–0.822)	2.110 ± 10.750	1.877 ± 0.899	0.955(0.938–0.967)	0.228	0.035	0.447
Family problems	0.839(0.799–0.873)	3.876 ± 3.110	4.969 ± 1.606	0.885(0.803–0.894)	1.055	0.230	2.923
Social support	0.843(0.799–0.879)	1.786 ± 0.985	1.778 ± 0.885	0.946(0.927–0.961)	0.229	0.050	0.635
Stress reaction	0.762(0.690–0.819)	1.271 ± 0.778	1.342 ± 0.707	0.898(0.861–0.925)	0.249	0.054	0.689
Family beliefs	0.783(0.723–0.833)	1.582 ± 0.912	1.631 ± 0.844	0.887(0.846–0.917)	0.306	0.067	0.849
Child problems	0.732(0.667–0.789)	7.152 ± 2.298	7.047 ± 1.752	0.978(0.970–0.984)	0.341	0.074	0.945
Sibling problems	0.770(0.718–0.819)	6.994 ± 3.648	7.585 ± 1.777	0.869(0.822–0.904)	1.320	0.288	3.660

SC-PAT 2.0 the Simplified Chinese version of The Psychological Assessment Tool

^a^ Data are reported as mean ± SD, ^b^ The MDC value at the group level, ^c^ The MDC value at the individual level

**Table 3:** The content validity, and construct validity of SC-PAT 2.0

	Floor effect (%)	Ceiling effect (%)	r of SC-PAT 2.0 with FAD-GF
Total	0(0)	0(0)	0.854^*^
Structure/resources	2(1.24)	0(0)	0.521^*^
Family problems	11(6.83)	3(1.86)	0.615^*^
Social support	1(0.62)	1(0.62)	0.400^*^
Stress reaction	6(3.73)	1(0.62)	0.555^*^
Family beliefs	1(0.62)	0(0)	0.523^*^
Child problems	0(0)	0(0)	0.370^*^
Sibling problems	1(0.62)	2(1.24)	0.337^*^

SC-PAT 2.0 the Simplified Chinese version of The Psychological Assessment Tool, FAD-GF the General Functioning Subscale of the Family Assessment Device, SD standard deviation, r Pearson’s correlation coefficient

^*^p < 0.05

### Reliability

**Fig. 2:**
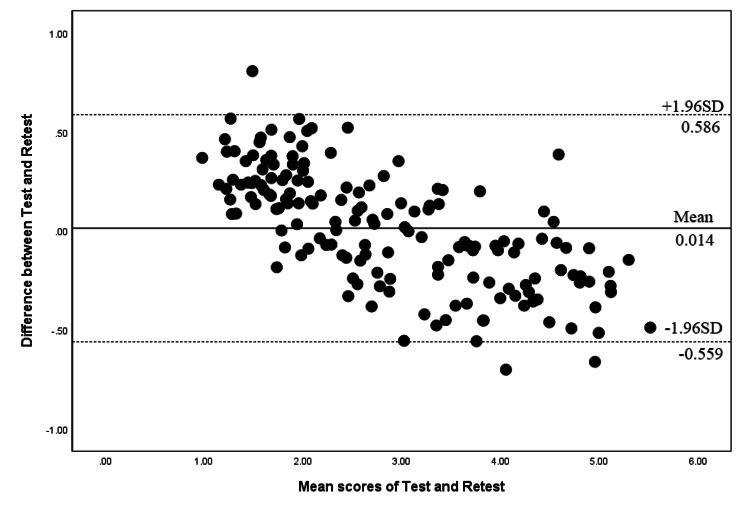
Bland–Altman plots of the test–retest reliability of the total score of SC-PAT 2.0. Each data point indicates how the difference between the two test sessions compares to the mean of the two sessions. The dashed line shows the 95% (± 1.96 SD) limits of agreement

The Cronbach’s α values for the total score and each of the 7 domains of SC-PAT2.0 range from 0.732 to 0.843, indicating acceptable internal consistency. The intraclass correlation coefficients (ICCs) for the total score and the 7 domains exceed 0.8, indicating good test-retest reliability of SC-PAT2.0 (Table 2). Figure 2 illustrates that the majority of data points in Bland-Altman plots fall within the 95% limit of agreement, confirming the absence of systematic errors in the two consecutive rounds of questionnaires. Table 2 also presents the SEM values and individual and group MDC values for each domain of SC-PAT2.0.

## Discussion

This study marks the inaugural exploration into the translation and validation of a Simplified Chinese Version of PAT2.0, focusing on assessing the family psychosocial risk of children with cancer. The most significant finding of the study is that SC-PAT2.0 demonstrates favorable score distribution, acceptable internal consistency, good test-retest reliability, notable content validity, and construct validity. The translation and validation of PAT2.0 not only facilitate intercultural communication and understanding but also expand the scope of PAT2.0 research, leading to increased sample sizes that enhance representativeness and reliability. Furthermore, this process contributes to the improved accessibility and popularity of mental health services. Such advancements play a crucial role in fostering the development and innovation of the mental health field. In conclusion, the importance of translating and validating research lies in promoting cross-cultural communication, broadening research horizons, enhancing mental health service accessibility, and contributing to the overall development of the mental health field.

The research findings reveal that all components in SC-PAT2.0 exhibit a Cronbach’s α value surpassing 0.7, indicating an acceptable level of correlation among them. Moreover, the Cronbach’s α value for the total score, family problems, and social support surpasses 0.8, signifying a substantial correlation among these specific items. Notably, the Cronbach’s α value in the Dutch version of the scale was lower than anticipated, potentially attributable to differences in the Dutch healthcare structure compared to countries where PAT2.0 has previously been validated [9]. Similarly, some components in the Japanese version of PAT2.0 display low Cronbach’s α values, likely stemming from alterations made by researchers based on Japanese cultural norms or the limited number of items per component [10]. Conversely, PAT2.0 entries in Turkish exhibit a robust correlation among all items [11].

The test-retest reliability of SC-PAT2.0 was assessed with an interval of 4–7 days, revealing good reliability. Four out of seven domains and the total score exhibited an ICC of > 0.9, while the other four domains also had an ICC value exceeding 0.9. Notably, prior studies have not explored the test-retest reliability of PAT2.0. MDC and SEM values indicate that SC-PAT2.0 is capable of detecting small clinical and individual-level changes. Additionally, the Bland-Altman plot for the total scores in both tests showed no systematic bias between the test and retest, affirming the good test-retest reliability of the total scale.

Concerning the construct validity of SC-PAT2.0, substantial correlations were observed between family problems and FAD-GF (0.615), while the correlations between child problems and FAD-GF (0.370) and sibling problems and FAD-GF (0.337) were deemed fair. Each subfield of SC-PAT2.0 demonstrated a modeled correlation with FAD-GF ranging from 0.400 to 0.555. Moreover, the total score of SC-PAT2.0 exhibited an almost perfect correlation with FAD-GF (0.854). The moderate correlation between child problems/sibling problems and FAD-GF may stem from the primary focus of these scales on children, whereas the significant correlation between family problems and FAD-GF emphasizes the scale’s effectiveness in assessing family function in children with cancer. This study marks the first exploration of the correlation between PAT2.0 and FAD-GF, highlighting the effectiveness of SC-PAT2.0 in this context.

Based on our results and data from other language versions, SC-PAT2.0 has effectively adapted to systematically and consistently identify psychosocial risk in families, displaying commendable scores across all indicators. This tool holds potential for providing evidence or education in nursing practice to enhance the quality of care for cancer patients. A notable limitation of this study is its single-center focus. In the next phase, we intend to collaborate with other children’s centers, increase participant enrollment, and conduct further testing and refinement of SC-PAT2.0.

## Conclusions

Following thorough translation and validation, we have successfully translated PAT2.0 into Chinese, ensuring accuracy, completeness of content, and user-friendliness, along with demonstrating good reliability, content validity, and structural validity. SC-PAT2.0 is not only suitable for Chinese-speaking audiences but also facilitates cross-cultural research. In summary, SC-PAT2.0 holds a crucial role in regularly screening children with cancer and their families for psychosocial problems. This can contribute to cross-cultural communication, broaden research horizons, improve the accessibility of mental health services, and foster the development of the mental health discipline.

## Data Availability

The data analyzed for the current study are available from the corresponding author on reasonable request.

## Abbreviations

CFA: Confirmatory factor analysis
CIs: Confidence intervals
CNS: Central Nervous System
FAD-GF: The General Functioning Subscale of the Family Assessment Device
ICC: The intra class correlation coefficient
KMO: Kaiser-Meyer-Olkin
MDC: The minimal detectable change
PAT 2.0: The Psychosocial Assessment Tool
SC-PAT 2.0: The Simplified Chinese version of The Psychological Assessment Tool
SD: Standard deviation
SEM: The standard error of measurement
